# Serum C-Reactive Protein (CRP) as a Simple and Independent Prognostic Factor in Extranodal Natural Killer/T-Cell Lymphoma, Nasal Type

**DOI:** 10.1371/journal.pone.0064158

**Published:** 2013-05-28

**Authors:** Ya-Jun Li, Zhi-Ming Li, Yi Xia, Jia-Jia Huang, Hui-Qiang Huang, Zhong-Jun Xia, Tong-Yu Lin, Su Li, Xiu-Yu Cai, Zhi-Jun Wu-Xiao, Wen-Qi Jiang

**Affiliations:** 1 State Key Laboratory of Oncology in South China, Guangzhou, China; 2 Department of Medical Oncology, Sun Yat-sen University Cancer Center, Guangzhou, China; 3 Department of Hematological Oncology, Sun Yat-sen University Cancer Center, Guangzhou, China; University of Nebraska Medical Center, United States of America

## Abstract

**Background:**

C-reactive protein (CRP) is a biomarker of the inflammatory response, and it shows significant prognostic value for several types of solid tumors. The prognostic significance of CRP for lymphoma has not been fully examined. We evaluated the prognostic role of baseline serum CRP levels in patients with extranodal natural killer (NK)/T-cell lymphoma (ENKTL).

**Methods:**

We retrospectively analyzed 185 patients with newly diagnosed ENKTL. The prognostic value of the serum CRP level was evaluated for the low-CRP group (CRP≤10 mg/L) versus the high-CRP group (CRP>10 mg/L). The prognostic value of the International Prognostic Index (IPI) and the Korean Prognostic Index (KPI) were evaluated and compared with the newly developed prognostic model.

**Results:**

Patients in the high-CRP group tended to display increased adverse clinical characteristics, lower rates of complete remission (*P*<0.001), inferior progression-free survival (PFS, *P* = 0.001), and inferior overall survival (OS, *P*<0.001). Multivariate analysis demonstrated that elevated serum CRP levels, age >60 years, hypoalbuminemia, and elevated lactate dehydrogenase levels were independent adverse predictors of OS. Based on these four independent predictors, we constructed a new prognostic model that identified 4 groups with varying OS: group 1, no adverse factors; group 2, 1 factor; group 3, 2 factors; and group 4, 3 or 4 factors (*P*<0.001). The novel prognostic model was found to be superior to both the IPI in discriminating patients with different outcomes in the IPI low-risk group and the KPI in distinguishing between the low- and intermediate-low-risk groups, the intermediate-low- and high-intermediate-risk groups, and the high-intermediate- and high-risk groups.

**Conclusions:**

Our results suggest that pretreatment serum CRP levels represent an independent predictor of clinical outcome for patients with ENKTL. The prognostic value of the new prognostic model is superior to both IPI and KPI.

## Introduction

Extranodal natural killer (NK)/T-cell lymphoma (ENKTL), nasal type, is a distinct and heterogeneous histopathologic subtype of non-Hodgkin lymphoma (NHL) characterized by vascular damage and destruction, prominent necrosis, and association with Epstein-Barr virus (EBV) [1]. The frequency of ENKTL among NHL patients is significantly higher in Asia than in Western countries [2], [3]. Despite radiotherapy and chemotherapy, the prognosis for ENKTL patients is poor, with 5-year survival rates ranging from 32% to 49.5% [2]–[5]. The poor prognosis and significant heterogeneity of ENKTL emphasize the need for more efficient prognostic factors or models to stratify patients with different survival outcomes. Although the prognostic value of the International Prognostic Index (IPI) has been well validated for many subtypes of NHL, its prognostic significance for ENKTL remains controversial [3]–[7]. Recently, a new prognostic model, the Korean Prognostic Index (KPI), which is specific for ENKTL, nasal type, has been proposed, and its prognostic value has been verified by several studies [3]–[5]. The KPI model may be further improved by other laboratory-based parameters (i.e., C-reactive protein, hemoglobin, platelet count, and albumin levels) and pathologic data [3], [5].

C-reactive protein (CRP) is an acute-phase protein secreted by hepatocytes during the inflammatory response, and it is regulated by pro-inflammatory cytokines [8]. Multiple studies have demonstrated that elevated serum CRP levels are associated with poor prognosis for various solid tumors, such as esophageal cancer [9], colorectal cancer [10], hepatocellular carcinoma [11], renal cell carcinoma [12], breast cancer [13], and lung cancer [14]. Serum CRP levels also represent a valuable prognostic variable in Hodgkin lymphoma (HL) and aggressive NHL [3], [15]–[18]. The prognostic value of serum CRP levels for ENKTL has not been fully examined. Therefore, we designed this study to evaluate the prognostic significance of serum CRP levels in ENKTL and develop a new prognostic model.

## Materials and Methods

### Ethics Statement

Written informed consent for patients’ blood samples and other medical information to be stored in our hospital database were obtained from all patients, and we also obtained separate consent for use of research. This study was approved by the Institutional Review Board of the National Cancer Institute, as well as ethics committees of Sun Yat-Sen University Cancer Center. The study was performed in accordance with the Declaration of Helsinki and the institutional guidelines of the local ethics committee.

### Patient Selection

We performed a retrospective study of 185 consecutive patients with newly diagnosed ENKTL, nasal type, at the Sun Yat-sen University Cancer Center between October 2006 and January 2012. All patients included in this study met the following criteria: (a) Pathologically confirmed diagnosis of ENKTL, nasal type, by expert pathologists, according to the WHO classification [1]. (b) No previous malignancy or any second primary tumor and no previous anti-cancer treatment. (c) Available data on baseline serum CRP levels. (d) Adequate clinical, laboratory, and follow-up data. (e) Patients with blastic NK-cell lymphoma/leukemia, aggressive NK-cell lymphoma/leukemia, or peripheral T-cell lymphoma, unspecified, were excluded. (f) Patients with any clinical evidence of acute infection or chronic active inflammatory disease, such as rheumatoid arthritis, were also excluded.

Before treatment, the following baseline clinical data were collected: patient demographics, physical examinations, Eastern Cooperative Oncology Group performance status (ECOG PS), primary site, B symptoms, treatment modalities and response, blood cell count, serum lactate dehydrogenase (LDH), baseline serum CRP levels, serum Epstein–Barr virus-DNA (EBV-DNA) copy number, Ann Arbor stage, bone marrow status, and computed tomography (CT) or magnetic resonance (MR) image of the nasopharynx, neck, chest, abdomen, and pelvis or positron emission tomography/computed tomography (PET/CT) of the entire body. All patients were staged using the Ann Arbor staging system. The IPI (age, ECOG PS, stage, LDH level, extranodal sites) and KPI for nasal NK/T-cell lymphoma (stage, LDH level, B symptoms, regional lymphoma nodes) were also used to perform survival analysis [4], [19]. ENKTL was divided into two subtypes: upper aerodigestive tract NK/T-cell lymphoma (UNKTL) and extra-upper aerodigestive tract NK/T-cell lymphoma (EUNKTL) according to the definitions given in previous studies [3], [4]. We also collected data relevant to comorbidities, such as diabetes, cardiovascular disease, chronic hepatitis, smoking, and hypertension, as previous studies have indicated that these factors promote increased serum CRP levels [8], [20]. The definitions of these comorbidities are as follows: chronic hepatitis B: HBsAg-positive >6 months and serum HBV-DNA >2000 IU/ml (10^4^ copies/ml) with or without elevation in alanine transaminase/aspartate transaminase levels; hypertension: systolic blood pressure ≥140 mmHg, diastolic blood pressure ≥90 mmHg, or a previous diagnosis of hypertension by a healthcare professional; diabetes: fasting plasma glucose level ≥7.0 mmol/L, and/or 2-h plasma glucose level ≥11.1 mmol/L after a 75 g glucose load, or a previous diagnosis of diabetes by a healthcare professional; cardiovascular disease: includes coronary heart disease, cerebrovascular disease, peripheral arterial disease, rheumatic heart disease, congenital heart disease, and deep vein thrombosis and pulmonary embolism, or a previous diagnosis of above diseases by a healthcare professional; smoking: those patients who smoked at the time of the diagnosis and who had smoked at least 100 cigarettes during their lifetime.

### Serum CRP Level Measurement

Baseline serum CRP level measurements were included in the routine clinical tests via a modified latex-enhanced immunoturbidimetric assay using a CRP latex kit (HITACHI 7600-020) according to the manufacturer’s instructions.

### Response Criteria and Statistical Analysis

The response to treatment was assessed according to the International Working Group Recommendations for Response Criteria for non-Hodgkin lymphoma [21]. Progression-free survival (PFS) was defined as the interval between the date of diagnosis and the date of first relapse, progression, death from any cause, or the last date at which patients were censored. Overall survival (OS) was defined from the date of diagnosis until either the time of death from any cause or the last date at which patients were censored. Receiver-operating-characteristics (ROC) analysis was used to determine an optimal cutoff for CRP concentration in predicting disease progression or death. Categorical characteristics were compared using a chi-square test. The log-rank test and Kaplan-Meier method were applied for univariate survival analysis. Variables significant at *P*<0.05 in univariate analysis were included in multivariate analysis. Multivariate analysis was performed according to the Cox proportional hazards model. A two-tailed *P*-value <0.05 was considered statistically significant. The statistical software package SPSS 16.0 (SPSS, USA) was used for statistical calculations.

## Results

### Patient Characteristics

In total, 185 patients (125 male, 60 female; median age, 43 years [range 18–71]) met the inclusion criteria. The clinical characteristics of the 185 patients are listed in Table 1. Most patients (182 cases, 98.4%) displayed a favorable performance status (ECOG PS 0–1). Ninety-two patients (49.7%) presented with B symptoms. Elevated LDH levels were observed for 53 cases (28.6%). Twenty-six patients (14.1%) had a mass ≥5 cm, and only 4 patients (2.2%) displayed bone marrow involvement. Seventy-three patients (39.5%) displayed regional lymph node involvement, and 26 patients (14.1%) displayed extranodal involvement sites ≥2. Most patients (161 cases, 87.0%) had localized disease (stage I/II). The disease diagnosis was UNKTL for 170 patients (91.9%) and EUNKTL in only 15 patients (8.1%). According to the IPI, a majority of the patients (156 cases, 84.3%) were classified as low/low-intermediate risk (IPI = 0–2), and 29 patients (15.7%) were categorized as intermediate-high/high risk (IPI = 3–5). The number of patients with KPI = 0–1 (110 cases, 59.5%) was significantly higher than those with KPI = 2–4 (75 cases, 40.5%).

**Table 1:** Baseline characteristics of patients by serum CRP level.

Characteristics	Mean CRP (mg/L)	*P*	Low-CRP group, n (%)	High-CRP group, n (%)	*P*
No. of cases			110 (59.5)	75 (40.5)	
Age (median [range], years)		0.425	43 (18–70)	43 (19–71)	0.001
≤60	14.83±24.48		101 (91.8)	56 (74.7)	
>60	18.99±18.35		9 (8.2)	19 (25.3)	
Gender (male)	15.88±23.71	0.675	70 (63.6)	55 (73.3)	0.167
ECOG PS		0.097			0.737
0–1	15.00±23.31		109 (99.1)	73 (97.3)	
≥2	37.97±44.43		1 (0.9)	2 (2.7)	
B symptoms (Yes)	22.22±29.75	<0.001	39 (35.5)	53 (70.7)	<0.001
LDH >245 U/l	24.06±30.33	0.009	22 (20.0)	31 (41.3)	0.002
Mass ≥5 cm	18.34±21.27	0.494	13 (11.8)	13 (17.3)	0.289
Extranodal sites ≥2	18.50±22.91	0.470	15 (13.6)	11 (14.7)	0.843
Regional LN involvement	17.22±26.54	0.393	40 (36.4)	33 (44.0)	0.297
EBV-DNA^a^ (median [range], copies/ml)		0.073	2,505 (0–20,000,000)	7,470 (0–50,000,000)	0.141
<5,050	10.61±12.29		26 (56.5)	14 (40.0)	
≥5,050	20.58±32.54		20 (43.5)	21 (60.0)	
Subtype		0.553			0.965
UNKTL	15.06±23.58		101 (91.8)	69 (92.0)	
EUNKTL	18.87±26.54		9 (8.2)	6 (8.0)	
Ann Arbor stage		0.290			0.145
I/II	14.65±23.59		99 (90.0)	62 (82.7)	
III/IV	20.17±24.l95		11 (10.0)	13 (17.3)	
Comorbidities					
Chronic hepatitis B	17.71±23.21	0.670	10 (9.1)	7 (9.3)	0.955
Hypertension	10.40±19.51	0.522	7 (6.4)	2 (2.7)	0.424
Diabetes	9.00±6.00	0.506	4 (3.6)	2 (2.7)	1.000
Cardiovascular disease	3.10±0.00	0.606	1 (0.9)	0(0)	1.000
Smoking	20.14±28.05	0.180	20 (18.2)	16 (21.3)	0.595
IPI score		0.260			0.182
0–1	14.52±23.55		96 (87.3)	60 (80.0)	
2–5	19.95±24.87		14 (12.7)	15 (20.0)	
KPI score		0.001			<0.001
0–1	9.71±13.46		77 (70.0)	33 (44.0)	
2–4	23.67±31.96		33 (30.0)	42 (56.0)	
Albumin (<35 g/L)	39.79±41.99	0.005	7 (6.4)	20 (26.7)	<0.001
Leukocytes (<4 × 10^9^/L)	11.91±13.89	0.460	13 (11.8)	9 (12.0)	0.970
Hemoglobin (<110 g/L)	39.41±44.42	0.014	7 (6.4)	13 (17.3)	0.018
Platelets (<150 × 10^9^/L)	21.69±23.24	0.164	10 (9.1)	14 (18.7)	0.057

a Data of EBV-DNA copy number were available for 81 patients and the median value was 5,050 copies/ml.

Abbreviations: CRP: C-reactive protein; ECOG PS: Eastern Cooperative Oncology Group performance status; LDH: lactate dehydrogenase; LN: lymph node; EBV-DNA: Epstein–Barr virus-DNA; UNKTL: upper aerodigestive tract NK/T-cell lymphoma; EUNKTL: extra-upper aerodigestive tract NK/T-cell lymphoma; IPI: International Prognostic Index; KPI: Korean Prognostic Index.

### Baseline Serum CRP Levels

The median value for the baseline CRP levels in all patients was 6.27 mg/L (range: 0.16–154.92 mg/L). We performed a ROC curve analysis to determine the CRP cutoff to distinguish the two groups and found that the optimal cutoff for CRP level was 9.81 mg/L. Since a CRP level ≤8.02 mg/L was defined as the normal range in our center, and 10 mg/L was applied as the cutoff value for most of the previous studies and CRP concentrations >10 mg/L indicate a systemic inflammatory response [9]–[14], [20], we evaluated the prognostic value of these different CRP level cutoff points, including >6.27 mg/L (median value), and CRP levels >10 mg/L were found to be the most discriminatory threshold value with the smallest *P* value (*P*<0.001, other data not shown). Based on the ROC analysis result and these above findings, we used the CRP level >10 mg/L as the cutoff value in the present study. We defined the patients with serum CRP levels ≤10 mg/L as the low-CRP group and patients with serum CRP levels >10 mg/L as the high-CRP group. Based on this classification, 110 patients (59.5%) were categorized into the low-CRP group (≤10 mg/L), and 75 patients (40.5%) were categorized into the high-CRP group (>10 mg/L). The baseline clinical features of the patients in the low-CRP group were compared with those in the high-CRP group (Table 1). The high-CRP group was characterized by a higher proportion of patients with age >60 years, more frequent B symptoms, elevated LDH levels, elevated KPI scores, hypoalbuminemia, and anemia. No significant intergroup differences in other clinical characteristics were observed between the low-CRP and high-CRP groups (Table 1). Additionally, no significant differences in the incidence of comorbidities capable of influencing CRP levels, such as chronic hepatitis B, hypertension, diabetes, cardiovascular disease, and smoking, were observed between these two groups. No significant difference in the mean ± standard deviation (SD) serum CRP levels was observed between patients with one or more of the five comorbidities compared with patients lacking any comorbidities (17.46±25.61 vs. 14.34±22.85 mg/L, respectively, *P* = 0.403).

### Treatment Modalities and Response

The primary treatment modalities were as follows: (a) 111 cases (60.0%) received chemotherapy followed by radiotherapy (RT); (b) 54 cases (29.2%) received chemotherapy alone; (c) 4 cases (2.2%) received radiotherapy alone; (d) 8 cases (4.3%) received surgery followed by chemotherapy; and (e) 8 cases (4.3%) received only best supportive care. The treatment details and responses are listed in Table 2. No significant difference was found in the treatment modalities between the patients with CRP levels ≤10 mg/L compared with patients displaying CRP levels >10 mg/L (*P*>0.05). After the initial treatment, 125 of the 177 treated patients (70.6%) displayed a complete response (CR) or CR unconfirmed (CRu). The rate of CR to initial treatment was significantly higher in the low-CRP group than in the high-CRP group (82.7% vs. 53.4%, respectively, *P*<0.001).

**Table 2:** Primary treatment and response in patients with extranodal natural killer (NK)/T-cell lymphoma.

Treatment	Low-CRP group, n (%)	High-CRP group, n (%)	*P*
Patients treated	104 (94.5)	73 (97.3)	0.584
Treatment modalities			0.092
CT followed by RT	71 (64.5)	40 (53.3)	
CT alone	24 (21.8)	30 (40.0)	
RT alone	3 (2.7)	1 (1.3)	
Surgery followed by CT	6 (5.5)	2 (2.7)	
Best supportive care	6 (5.5)	2 (2.7)	
Chemotherapy regimens			0.986
CHOP or CHOP-like	12 (11.9)	8 (11.1)	
EPOCH	38 (37.6)	25 (34.7)	
ATT	22 (21.8)	17 (23.6)	
GEMOX+L-asp	27(26.7)	21 (29.2)	
SMILE	2 (2.0)	1 (1.4)	
Complete remission	86 (82.7)	39 (53.4)	<0.001

Abbreviations: CRP: C-reactive protein; CT: chemotherapy; RT: radiotherapy; CHOP: cyclophosphamide+doxorubicin+vincristine+prednisone; EPOCH: etoposide+doxorubicin+vincristine+cyclophosphamide+prednisone; ATT: alternating triple therapy (CHOP-B, cyclophosphamide+doxorubicin+vincristine+bleomycin+prednisone; IMVP-16, ifosfamide+methotrexate+etoposide; DHAP, dexamethasone+cisplatin+cytarabine); GEMOX+L-asp: gemcitabine+oxaliplatin+L-asparaginase; SMILE: dexamethasone+methotrexate+ifosfamide+L-asparaginase+etoposide.

### Survival and Prognostic Factors

**Figure 1:**
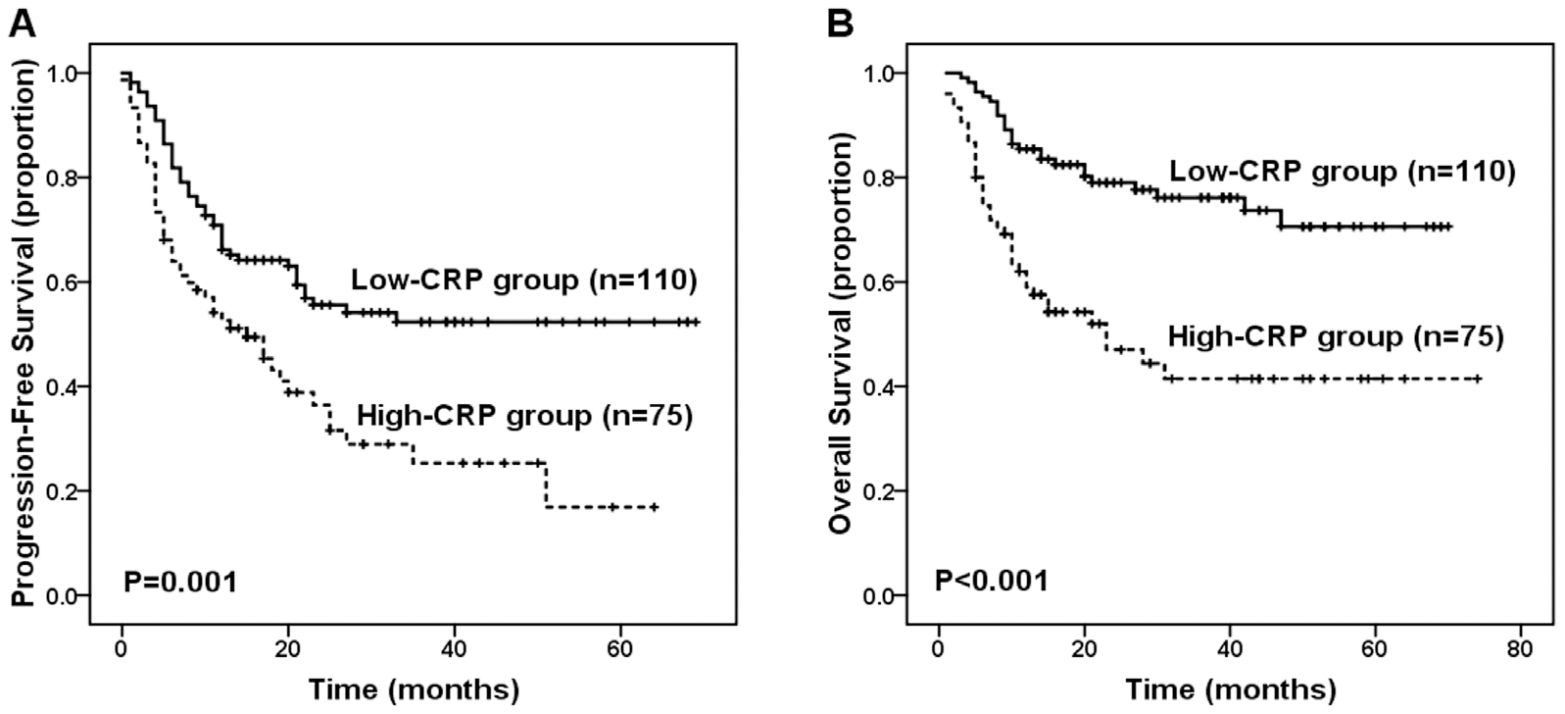
Survival outcome of patients based on the C-reactive protein (CRP) level. (A) Progression-free survival (PFS) of patients according to baseline CRP level (≤10 mg/L vs. >10 mg/L). (B) Overall survival (OS) of patients according to baseline CRP level (≤10 mg/L vs. >10 mg/L).

There were 64 deaths (34.6%) during a median follow-up of 31 months (range, 5–71 months), and all except 3 deaths were due to tumor progression. The estimated 3-year PFS and OS rates for all 185 patients were 42.2% and 62.8%, respectively. Patients in low-CRP group had significantly better PFS (3-year PFS, 52.3% vs. 25.3%, respectively; *P* = 0.001, Figure 1A) and OS (3-year OS, 76.1% vs. 41.5%, respectively; *P*<0.001, Figure 1B). For patients receiving chemotherapy followed by radiotherapy (111 cases, 60%), elevated serum CRP levels were significantly associated with inferior OS (3-year OS, 57.0% vs. 83.0%; *P* = 0.029) and inferior PFS with borderline significance (3-year PFS, 35.0% vs. 57.1%; *P* = 0.076).

Table 3 displays the results of the univariate and multivariate analysis of the potential predictors of PFS and OS. Multivariate analysis using the forward conditional Cox region model identified CRP levels >10 mg/L (RR = 1.924, 95% CI: 1.284–2.883, *P = *0.002) and an IPI score ≥2 (RR = 2.678, 95% CI: 1.648–4.354, *P*<0.001) as two adverse factors for PFS. In the multivariate analysis for OS, age >60 years (RR = 3.523, 95% CI: 1.857–6.682, *P*<0.001), elevated LDH levels (RR = 2.741, 95% CI: 1.592–4.717, *P*<0.001), CRP levels >10 mg/L (RR = 1.952, 95% CI: 1.158–3.293, *P* = 0.012), and albumin <35 g/L (RR = 2.851, 95% CI: 1.525–5.330, *P* = 0.001) were found to be significant independent predictors of OS.

**Table 3:** Univariate and multivariate analysis of prognostic factors for PFS and OS in patients with ENKTL.

Factors	PFS			OS		
	Univariate analysis	Multivariate analysis		Univariate analysis	Multivariate analysis	
	*P*	RR (95% CI)	*P*	*P*	RR (95% CI)	*P*
Age >60 years	0.190			0.001	3.523 (1.857–6.682)	<0.001
B symptoms	0.042			0.013		
Mass ≥5 cm	0.031			0.051		
Extranodal sites ≥2	0.003			0.041		
Regional LN involvement	0.042			0.201		
Stage III/IV	<0.001			0.002		
Subtype, EUNKTL	0.002			0.085		
LDH >245 U/l	0.001			<0.001	2.741 (1.592–4.717)	<0.001
CRP>10 mg/L	0.001	1.924 (1.284–2.883)	0.002	<0.001	1.952 (1.158–3.293)	0.012
Albumin <35 g/L	0.007			<0.001	2.851 (1.525–5.330)	0.001
IPI score ≥2	<0.001	2.678 (1.648–4.354)	<0.001	<0.001		
KPI score ≥2	<0.001			<0.001		
ALC <1 × 10^9^/L	0.049			0.032		
Hemoglobin <110 g/L	0.193			0.131		
Platelets <150 × 10^9^/L	0.038			0.070		

Abbreviations: PFS: progression-free survival; OS: overall survival; LN: lymph node; ENKTL: extranodal NK/T-cell lymphoma; RR: relative risk; CI: confidence interval; EUNKTL: extra-upper aerodigestive tract NK/T-cell lymphoma; LDH: lactate dehydrogenase; CRP: C-reactive protein; IPI: International Prognostic Index; KPI: Korean Prognostic Index; ALC: absolute lymphocyte count.

Since CRP level was significantly associated with B symptoms, LDH level and age, a Cox region model analysis only including these four parameters was also performed to evaluate whether the CRP level was associated with PFS and OS independent of these variables. Multivariate analysis demonstrated that the CRP level remained a significant independent predictor of PFS (RR = 1.797, 95% CI: 1.170–2.760, *P* = 0.007) and OS (RR = 2.199, 95% CI: 1.312–3.686, *P* = 0.003), and the LDH level and age remained independent prognostic factors for OS (*P*<0.001 and = 0.001, respectively). Given that age and LDH level were two predictors of outcome independent of the CRP level, they were included in the following novel prognostic model.

### Clinical Difference between UNKTL and EUNKTL

Since previous studies have reported that EUNKTL was associated with adverse clinical features and poor prognosis [4], [22], we performed an analysis to compare the clinical features and survival outcome of UNKTL and EUNKTL in the present cohort. The EUNKTL group had a significant higher proportion of patients with elevated LDH levels, mass ≥5 cm, extranodal sites ≥2, advanced stage (stage III/IV), IPI score ≥2, hypoalbuminemia, and thrombocytopenia than the UNKTL group. Moreover, the EUNKTL group showed a lower complete remission rate (33.3% vs. 70.6%, *P* = 0.008) and inferior PFS rate (3-year PFS: 17.8% vs. 44.5%, *P* = 0.002) than the UNKTL group. However, no significant intergroup differences in other clinical characteristics and OS rate (3-year OS: 42.0% vs. 64.7%, *P* = 0.085) were observed between the EUNKTL and UNKTL groups. In addition, EUNKTL was not an independent predictor for inferior PFS and OS in multivariate analysis (Table 3).

### Prognostic Model

**Figure 2:**
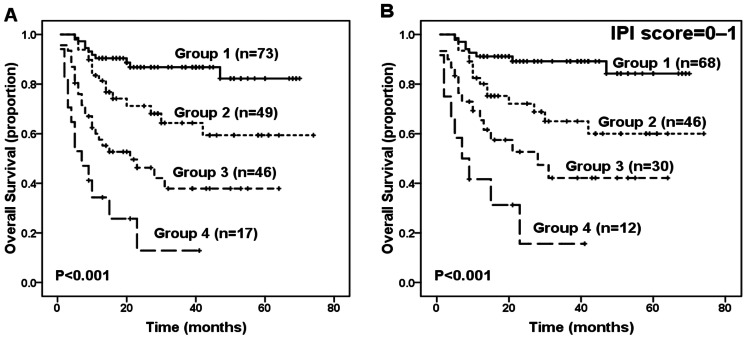
Survival outcome of patients according to the novel prognostic model. (A) Overall survival (OS) according to the new prognostic index for patients with extranodal natural killer (NK)/T-cell lymphoma (ENKTL), nasal type. (B) OS of patients with low International Prognostic Index risk (IPI score = 0–1) according to the new prognostic index.

Based on these 4 independent prediction factors (age >60 years, elevated LDH, CRP>10 mg/L, and albumin <35 g/L) for OS in the multivariate analysis, we constructed a new prognostic model by combing these prognostic variables as follows: group 1 (73 cases, 39.5%), no adverse factors; group 2 (49 cases, 26.5%), 1 factor; group 3 (46 cases, 24.9%), 2 factors; and group 4 (17 cases, 9.2%), 3 or 4 factors. The proportion of patients in each group and the associated hazard ratios are presented in Table 4. This novel prognostic model enabled the efficient identification of 4 groups of patients with different outcomes (*P*<0.001, Figure 2A). Furthermore, significant differences in OS were found between group 1 and group 2 (*P* = 0.010), between group 2 and group 3 (*P = *0.010), and between group 3 and group 4 (*P* = 0.022). The 3-year OS was 86.8% for group 1, 64.3% for group 2, 37.9% for group 3, and 12.9% for group 4.

**Table 4:** Overall survival and relative risk of death according to risk group as defined by the new prognostic index.

Risk group	No. of factors^a^	No. of patients (%)	3-year OS (%)	RR (95% CI)
Group 1	0	73 (39.5)	86.8	1.0 (N/A)
Group 2	1	49 (26.5)	64.3	2.7 (1.2–5.8)
Group 3	2	46 (24.9)	37.9	5.8 (2.8–12.2)
Group 4	3–4	17 (9.2)	12.9	13.3 (5.8–30.7)

a Factors: age >60 years, LDH >245 U/l, CRP>10 mg/L, and albumin <35 g/L.

Abbreviations: OS: overall survival; RR: relative risk; CI: confidence interval; N/A: not applicable.

Using the IPI predictive model, we identified 3 categories of patients with different survival outcomes: low risk (IPI = 0–1), 156 patients (84.3%); intermediate risk (IPI = 2–3), 25 patients (13.5%), and high risk (IPI = 4–5), 4 patients (2.2%). The 3-year OS was 67.7% for the low-risk group, 39.6% for the intermediate-risk group, and 25% for the high-risk group (*P*<0.001). Significant differences in survival were also found between the low-risk and intermediate-risk groups (*P* = 0.005) as well as between the intermediate-risk and high-risk groups (*P* = 0.018). However, based on the IPI data, 84.3% of the patients were disproportionately grouped into the low-risk group, and the IPI score was unable to identify patients with different survival statuses within the low-risk group. The novel prognostic model efficiently categorized patients in the low-risk IPI group into four subgroups with different survival outcomes (*P*<0.001, Figure 2B).

**Figure 3:**
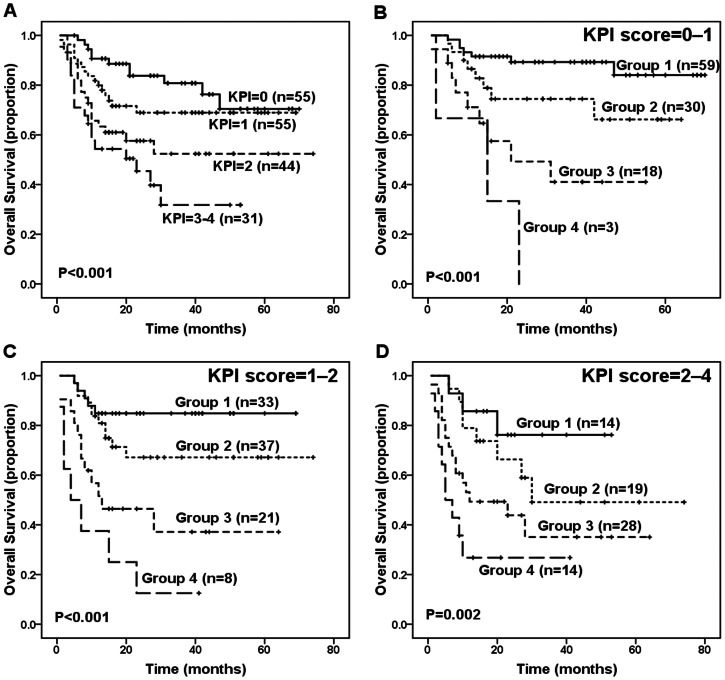
Survival outcome of patients according to the Korean Prognostic Index (KPI) score. (A) Overall survival (OS) according to the KPI for patients with extranodal natural killer (NK)/T-cell lymphoma (ENKTL), nasal type. (B) OS of patients with KPI score = 0–1 according to the new prognostic index. (C) OS of patients with KPI score = 1–2 according to the new prognostic index. (D) OS of patients with KPI score = 2–4 according to the new prognostic index.

The KPI model balanced the distribution of patients in different risk categories more efficiently than the IPI model (score 0∶55 cases, 29.7%; score 1∶55 cases, 29.7%; score 2∶44 cases, 23.8%; and score 3–4∶31 cases, 16.8%), and it was able to discriminate between patients with different survival outcomes (*P*<0.001, Figure 3A). However, the KPI model failed to significantly distinguish between the low- and intermediate-low-risk groups (*P* = 0.198), the intermediate-low- and high-intermediate-risk groups (*P* = 0.119), and the high-intermediate- and high-risk groups (*P* = 0.233). In contrast, the new prognostic index was found to efficient in discriminating patients with KPI score = 0–1 (*P*<0.001, Figure 3B) or patients with KPI score = 1–2 (*P*<0.001, Figure 3C) or patients with KPI score = 2–4 (*P* = 0.002, Figure 3D). The pros and cons of the IPI, KPI and the current new model are summarized in Table 5.

**Table 5:** The pros and cons of the IPI, KPI and current prognostic model.

Charactristics	IPI	KPI	Current model
Parameters	Age >60 years	LDH >normal	Age >60 years
	LDH >normal	B symptoms	LDH >normal
	ECOG PS = 2–4	Regional LN involvement	CRP>10 mg/L
	Stage III/IV	Stage III/IV	Albumin <35 g/L
	Extranodal sites ≥2		
Risk groups	Low (score = 0–1)	Low (score = 0)	Group 1 (score = 0)
	Intermediate (score = 2–3)	Low intermediate (score = 1)	Group 2 (score = 1)
	High (score = 4–5)	High intermediate (score = 2)	Group 3 (score = 2)
		High (score = 3–4)	Group 4 (score = 3–4)
Pros	**a.** Have been widely validated in manyB-cell subtypes of NHL	**a.** Balance the distribution of patients in different risk groups better than theIPI and current model	**a.** Incorporates both the indicators of the inflammatory response and tumor burden
	**b.** Can efficiently distinguish 3 groups of patients with different overall survival	**b.** Can efficiently distinguish 4 groups of patients with different overall survival	**b.** Can efficiently distinguish 4 groups of patients with different overall survival and can efficiently distinguish patients in the low risk IPI group
	**c.** Can significantly discriminate between the adjacent two risk groups	**c.** Have been validated in external cohort	**c.** Can significantly discriminate between the adjacent two risk groups
Cons	**a.** Proportion of patients in each risk groupis unequal and 84.3% of the patientsare allocated in low risk group	**a.** Failed to significantly discriminate between the neighboring two risk groups	**a.** Have not been validated in an external large sample of cohort
	**b.** Failed to distinguish patients within thelow risk group		

Abbreviations: IPI: International Prognostic Index; KPI: Korean Prognostic Index; LDH: lactate dehydrogenase; ECOG PS: Eastern Cooperative Oncology Group performance status; LN: lymph nodes; CRP: C-reactive protein.

## Discussion

CRP is an essential biomarker of the inflammatory response [8]. Several studies have indicated that serum CRP levels are an independent predictor of prognosis for various solid tumors, HL, and NHL [3], [9]–[18]. However, the prognostic role of serum CRP levels in ENKTL remains unclear. The distinct characteristics of prominent regional necrosis and inflammation observed in ENKTL patients prompted us to analyze the impact of CRP on the survival outcome of patients with ENKTL. To the best of our knowledge, the present study represents the largest series and the second one to date examining the prognostic value of serum CRP levels in ENKTL.

The results of the current study are consistent with the recent study of Au et al., in which serum CRP levels were first reported as a significant predictor of prognosis for ENKTL patients [3]. However, in that study, the number of patients who had data on serum CRP concentrations was small (only 64 cases), and the report failed to clearly describe the cutoff value for CRP. Several different CRP concentration cutoff points, including >5 mg/L, >3.9 mg/L and >10 mg/L, have been used in previous studies involving solid tumors [9]–[14]. Regarding lymphomas, a study on HL by Wieland et al. and another study on NHL reported by Herishanu et al. used 5 mg/L as the cutoff point for CRP levels [15], [17]. However, another study on NHL by Legouffe et al. defined a serum value >10 mg/L as elevated CRP levels [16]. As a CRP level ≤8.02 mg/L was defined as the normal range in our center, a more recent study from our center used CRP levels >8.02 mg/L as a cutoff value and found that elevated serum CRP levels were significantly associated with a poor prognosis in diffuse large B-cell lymphoma (DLBCL) patients [18]. In the present study, CRP levels >10 mg/L were found to be the most discriminatory threshold value among different CRP level cutoff, and it was very close to the optimal cutoff identified by ROC analysis. Moreover, several previous studies have revealed that CRP levels >10 mg/L are likely to indicate a system inflammatory response and malignancies [9]–[14], [20]. Therefore, we adopted CRP levels >10 mg/L as the cutoff point in the present study. Despite the different cutoff values of CRP levels among various studies, elevated serum CRP levels are significantly associated with poor prognosis.

The mechanisms underlying the relationship between elevated serum CRP levels and poor prognosis are not clear; however, several potential explanations have been proposed. First, because various pro-inflammatory cytokines, such as interleukin-1 (IL-1), interleukin-6 (IL-6), tumor necrosis factor-α (TNF-α), IFN-γ, and tumor growth factor, all stimulate CRP production, survival, growth, proliferation, and the migration of tumor cells, elevated CRP levels may indirectly reflect the increased concentrations of these pro-inflammatory cytokines [8], [23]–[28]. Second, CRP affects tumor growth and survival by enhancing tumor cell proliferation and protecting tumor cells from drug-induced apoptosis [29]. If this is also the case in ENKTL, regulation of the inflammatory response and CRP may represent an important therapeutic target. Third, elevated CRP levels are associated with several adverse clinical features. Finally, as previous studies have shown that elevated CRP levels are associated with mortality within the general population [30], [31], the pretreatment baseline serum CRP levels may provide insight into the general health of patients at the time of diagnosis of ENKTL. Based on these findings and knowledge it is not surprising that elevated serum CRP levels are associated with poor prognosis.

Observations from previous studies of healthy individuals have shown that the serum CRP concentration is influenced by multiple factors including gender, race, age, body mass index (BMI), and lifestyles [32]–[35]. Increasing age, male, African American race (compared to non-Hispanic white), high BMI, and sedentary lifestyles are reported to be associated with increasing CRP levels, while weight loss, exercise training, and usage of anti-inflammatory drugs have been shown to reduce CRP levels in healthy individuals [32]–[35]. However, in the present study, there were no significant associations between age and gender and CRP levels in ENKTL patients. This implies that the factors affecting the CRP levels may be different between cancer patients and healthy population. Moreover, as our study focused on the Chinese lymphoma patients, who have not great heterogeneities in BMI and lifestyle, we believe that residual confounding due to lack of information on BMI and lifestyle is minor. Under pathological conditions, in addition to cancer, several comorbidities, including diabetes, cardiovascular disease, chronic hepatitis, smoking, and hypertension, have been reported to promote increases in serum CRP levels [8], [20]. However, in the present study, no significant between-group difference was observed in the CRP levels when the patients were divided into two groups: those with one or more of the above five comorbidities versus those without any comorbidities. We did not observe a significant difference in the incidence of these comorbidities between the low- and high-CRP groups. Taken together, the impact of possible confounding factors on serum CRP levels was excluded to a significant extent.

The prognostic value of the IPI score has been widely validated for DLBCL and many other subtypes of NHL. However, its prognostic role in ENKTL remains controversial [3], [4], [6], [7], [36]. In the present study, although IPI scores were significantly predictive in the univariate analysis, it failed to identify patients with varying survival rates within the low-risk group, which accounted for a majority of the patients. The KPI model yielded a balanced distribution of patients with different levels of risk and separated them into four groups with different survival outcomes. However, the KPI model failed to significantly distinguish between the neighboring two risk groups. These results confirm previously reported data [3]–[5], [36].

As ENKTL is frequently characterized by prominent necrosis and inflammation [1], it is rational to speculate that inflammation might play a crucial role in the prognosis of ENKTL. Furthermore, several recent studies have indicated that the Glasgow Prognostic Score (GPS), an inflammation-based cumulative prognostic score that evaluates only serum CRP and albumin levels, is one of the most useful prognostic models for a variety of common solid tumors [37]–[39]. Therefore, we aimed to develop a novel prognostic model for ENKTL that includes inflammatory biomarkers. Based on the four independent predictors, we constructed a new prognostic model for ENKTL. The prognostic value of the new prognostic model is superior to the IPI model and may be as effective as or better than KPI. As LDH is an indicator of tumor burden and CRP and albumin are markers of a systemic inflammatory response, the new prognostic score, which incorporates these three factors, reflects not only the tumor burden but also the host response. Additionally, the GPS score was found to be a powerful and independent predictor of survival outcome in patients with ENKTL in another study performed by our group (unpublished data). Based on these findings, it is plausible that the proposed prognostic model might have superior predictive value for the survival outcome compared with KPI for patients with ENKTL.

In conclusion, our study suggests that pretreatment baseline serum CRP levels are a significant and independent predictor of clinical outcome for ENKTL patients. The proposed prognostic model, which involves the inflammatory response marker CRP, may have superior predictive value for survival outcome compared with KPI for patients with ENKTL. Further studies are warranted to confirm the prognostic value of serum CRP levels and to determine whether the novel prognostic model can be used routinely to replace or improve the currently widely used KPI prognostic model in patients with ENKTL.
